# Comprehensive Proteomics and β-Hydroxybutyrylation Profiling in Starvation-Induced Gastrocnemius Muscle Remodeling

**DOI:** 10.3390/biology15030289

**Published:** 2026-02-06

**Authors:** Leilei Cui, Chunping Huang, Yu Su, Shiqi Xu, Liang Zha, Qiuyuan Zhao, Wu Quan, Xinqiang Lan, Yang Xiang, Qiquan Wang

**Affiliations:** 1 Metabolic Control and Aging, Human Aging Research Institute and School of Life Science, Nanchang University, Jiangxi Key Laboratory of Aging and Diseases, Nanchang 330031, China; leileicui_xuan@hotmail.com (L.C.); lanxinqiang@ncu.edu.cn (X.L.); 2 School of Life Sciences, Nanchang University, Nanchang 330031, China; 3 School of Public Health, Jiangxi Medical College, Nanchang University, Nanchang 330031, China; suyucll@163.com (Y.S.);; 4 School of Food Science and Technology, Nanchang University, Nanchang 330031, China

**Keywords:** starvation, skeletal muscle, lysine β-hydroxybutyrylation (Kbhb), proteomics, ketone body

## Abstract

Starvation elicits profound metabolic adaptations in skeletal muscle, enabling survival during nutrient scarcity. While global proteomic changes underpinning muscle atrophy have been studied, the role of lysine β-hydroxybutyrylation (Kbhb), a novel metabolite-derived post-translational modification linked to ketone metabolism, remains largely unexplored. In this study, we subjected mice to 72 h of food deprivation and performed integrative quantitative proteomics and Kbhb-modified peptide profiling on gastrocnemius muscle. Starvation induced significant body weight and muscle mass loss, accompanied by increased systemic β-hydroxybutyrate levels and widespread Kbhb modification of muscle proteins. Proteomic analysis revealed extensive downregulation of ribosomal and translation-associated proteins, coupled with upregulation of autophagy and lipid catabolism pathways, highlighting a coordinated shift from anabolic processes to catabolic and oxidative metabolism. Deep Kbhb profiling identified over 7500 modified lysine sites across 2000 proteins, with starvation triggering a global increase in Kbhb on key metabolic enzymes involved in glycolysis, TCA cycle, fatty acid β-oxidation, and amino acid metabolism. Notably, starvation-enhanced Kbhb preferentially targeted evolutionarily conserved lysines proximal to catalytic or cofactor-binding domains, implicating a regulatory role in enzymatic activity modulation. Conversely, Kbhb on structural and contractile proteins was downregulated, suggesting functional reprioritization of muscle physiology during fasting. Our findings uncover lysine β-hydroxybutyrylation as a dynamic, metabolically responsive PTM mediating gastrocnemius muscle adaptation to energy deficiency, expanding the paradigm of potentially metabolite-driven epigenetic and non-epigenetic regulatory mechanisms in muscle metabolism.

## Simple Summary

Starvation triggers profound metabolic reprogramming in skeletal muscle to sustain survival during nutrient scarcity, yet the role of lysine β-hydroxybutyrylation (Kbhb)—a metabolite-derived post-translational modification (PTM) linked to ketone metabolism—remains unclear. Here, we performed integrative quantitative proteomics and Kbhb profiling on gastrocnemius muscle from mice subjected to 72 h food deprivation. Starvation induced muscle atrophy, elevated systemic β-hydroxybutyrate levels, and widespread changes in Kbhb modification. Proteomic analysis revealed a shift from anabolic to catabolic/oxidative pathways, with downregulation of ribosomal proteins and upregulation of autophagy/lipid catabolism. Deep Kbhb profiling identified over 7500 modified sites across 2000 proteins, with starvation enhancing Kbhb on key metabolic enzymes (glycolysis, TCA cycle, fatty acid β-oxidation) at conserved residues proximal to functional domains, and reducing Kbhb on structural/contractile proteins. Our findings establish Kbhb as a dynamic PTM mediating gastrocnemius muscle adaptation to energy deficiency, expanding the role of metabolite-driven regulation in muscle metabolism.

## 1. Introduction

Skeletal muscle comprises a substantial proportion of body mass and plays a critical role in systemic energy homeostasis [1,2,3]. Nutrient deprivation, such as prolonged fasting, forces muscle to adapt by remodeling its metabolic and proteostatic networks to conserve energy, maintain function, and supply substrates for vital organs [4,5,6]. The muscle’s response to starvation includes muscle protein catabolism, activation of autophagy, and metabolic reprogramming favoring fatty acid oxidation and ketone utilization [7,8,9,10]. Despite advances, the molecular mechanisms coordinating these complex processes remain incompletely understood.

Post-translational modifications (PTMs) of proteins provide a rapid and reversible means to regulate protein function, localization, and interaction networks in response to metabolic cues [11,12,13,14,15]. Among these, lysine acylations, such as acetylation, succinylation, and malonylation, have emerged as key mediators linking cellular metabolism with protein function and gene regulation [16,17,18]. β-Hydroxybutyrylation (Kbhb), a recently identified lysine acylation derived from the ketone body β-hydroxybutyrate, was initially described as a histone mark associated with fasting and ketogenic states [19,20,21]. Subsequent studies have expanded its occurrence to non-histone proteins, implicating Kbhb in diverse biological processes [22,23,24]. However, the systemic landscape of Kbhb in skeletal muscle, its dynamic remodeling in response to acute nutrient deprivation, and its potential role in coordinating the muscle’s metabolic switch remain largely undefined.

Here, we present a comprehensive investigation of the skeletal muscle proteome and Kbhb modification landscape in a murine starvation model. The primary aims of this study were to: (i) systematically profile the global proteomic changes in skeletal muscle induced by acute starvation; (ii) establish a comprehensive landscape of lysine β-hydroxybutyrylation in this tissue; and (iii) investigate how starvation remodels the Kbhb proteome, with a particular focus on identifying modifications on key metabolic enzymes that may underlie the adaptive metabolic switch. Integrating quantitative proteomics with site-specific Kbhb profiling, we uncover extensive proteome remodeling and pervasive Kbhb dynamics that reflect and potentially mediate the metabolic switch from anabolic to catabolic states. Our structural and evolutionary analyses reveal that starvation-induced Kbhb preferentially targets functionally critical lysines on core metabolic enzymes, suggesting a mechanistic role in fine-tuning enzymatic activity during nutrient deprivation. These findings position Kbhb as a key regulatory PTM underlying skeletal muscle’s metabolic plasticity and highlight metabolic state-dependent PTM crosstalk as a fundamental layer of metabolic regulation.

## 2. Materials and Methods

### 2.1. Animals and Starvation Protocol

Male C57BL/6 mice were procured from Changsha Tianqin Biotechnology (Changsha, China). All experiments were performed using male C57BL/6 mice to minimize variability in the baseline metabolic and hormonal profiles. All experimental procedures adhered to ethical guidelines approved by the Institutional Animal Care and Use Committee of Nanchang University (protocol number: NCULAE-20221130019). Mice were randomly assigned to control or starvation groups, with animal age (8 weeks), initial weight range, acclimatization period (4 weeks), housing conditions (SPF, 12/12 light cycle), fasting procedure (only food removed in clean cages, water ad libitum), and precise sacrifice timing (between 9 and 11 AM). The starvation cohort underwent a 72 h fast with unrestricted access to water, while control animals were fed ad libitum. Baseline and post-starvation body weights were recorded. Post-experiment, gastrocnemius, tibialis anterior, and soleus muscles were rapidly excised, weighed, and flash-frozen in liquid nitrogen for subsequent analysis.

For animal studies, group allocation (control vs. starved) was performed by a researcher not involved in subsequent tissue harvesting and processing. Personnel involved in tissue dissection, sample preparation for mass spectrometry, and physiological data collection were blinded to group identity during these procedures.

### 2.2. Quantification of Circulating β-Hydroxybutyrate

Circulating β-hydroxybutyrate levels were measured from fresh tail vein whole blood using FreeStyle Precision β-ketone test strips (Abbott, Witney, UK), a method commonly used in murine studies of ketogenic metabolism and muscle ketone utilization as a semi-quantitative readout of systemic ketosis.

### 2.3. Western Blot Analysis

Muscle tissue lysates were prepared using RIPA buffer supplemented with a protease inhibitor cocktail (Yeasen, Shanghai, China). Protein concentrations were determined by the bicinchoninic acid (BCA) method (BioRAD, Hercules, CA, USA). Equal quantities of total protein per sample underwent SDS-PAGE and electrotransfer to PVDF membranes. After blocking with 5% BSA in PBS-Tween 20 (0.1%) at ambient temperature for 2 h, membranes were incubated overnight at 4 °C with primary antibodies against pan-Kbhb (PTM Bio, 1:1000, Hangzhou, China) and GAPDH (Proteintech, 1:2000, Wuhan, China). HRP-conjugated goat anti-mouse and goat anti-rabbit secondary antibodies (Servicebio, 1:5000, Wuhan, China) were applied subsequently at room temperature for 1 h. Chemiluminescence detection utilized SuperSignal reagents (Pierce, Rockford, IL, USA).

### 2.4. Sample Preparation for Proteome and β-Hydroxybutyrylation Enrichment

Frozen muscle tissues were pulverized under liquid nitrogen and lysed in 8 M urea buffer containing protease inhibitors, 3 μM trichostatin A, and 50 mM nicotinamide to preserve labile PTMs. Samples were sonicated on ice and centrifuged at 12,000× *g*, 4 °C for 10 min to clarify lysates. Proteins precipitated by 40% trichloroacetic acid were washed with acetone and resolubilized in 20% tetraethylammonium bromide buffer. After reduction with 5 mM dithiothreitol (56 °C, 30 min) and alkylation with 11 mM iodoacetamide (room temperature, 15 min, dark), samples were diluted to reduce urea concentration below 2 M. Trypsin digestion was performed overnight (enzyme:substrate ratio 1:50) followed by a supplementary digestion (1:100 ratio, 4 h). Peptides were desalted on C18 SPE columns prior to modification enrichment.

### 2.5. Affinity Enrichment of β-Hydroxybutyrylated Peptides

Peptides were resuspended in NETN buffer (100 mM NaCl, 1 mM EDTA, 50 mM Tris-HCl pH 8.0, 0.5% NP-40) and incubated with anti-Kbhb agarose-conjugated beads (20 μL; PTM-1201, PTM Bio) under gentle agitation at 4 °C overnight. After extensive washing with NETN and water, bound peptides were eluted with 0.1% trifluoroacetic acid, dried under vacuum, and desalted ahead of LC-MS/MS.

### 2.6. LC-MS/MS

Peptide samples were separated on a 25 cm × 75 μm reversed-phase C18 column using a Bruker nanoElute HPLC system (Bruker, Billerica, MA, USA). The gradient employed solvents A (0.1% formic acid, 2% acetonitrile) and B (0.1% formic acid in acetonitrile) with a flow rate of 450 nL/min: 6–24% B over 70 min, 24–35% in 14 min, ramping to 80% in 3 min and held for 3 min. Detection utilized a timsTOF Pro mass spectrometer operating in parallel accumulation serial fragmentation (PASEF) mode with an electrospray voltage of 1.60 kV. Data were acquired with an MS/MS scan range of 100–1700 *m*/*z*; up to 10 PASEF MS/MS scans were included per cycle, and dynamic exclusion was set to 30 s.

### 2.7. Data Processing and Quantitative Analysis

Raw files were processed with MaxQuant (v.1.6.15.0), searching against the reviewed Mus musculus SwissProt database along with a reversed decoy database for FDR estimation. Trypsin/P specificity was set, allowing four missed cleavages (proteome: two missed cleavages). Carbamidomethylation on cysteine residues was fixed; variable modifications included protein N-terminal acetylation, methionine oxidation, and lysine β-hydroxybutyrylation. Precursor and fragment mass tolerances were 20 ppm (first search)/5 ppm (main search) and 0.02 Da, respectively. Peptides ≥ 7 aa long with Kbhb localization probability ≥ 0.90 and FDR < 1% were considered.

Following database searching, stringent filtering was applied to ensure data quality. Proteins and Kbhb-modified peptides were retained only if they were identified with valid intensity values in at least three out of the four biological replicates within at least one experimental group. To enable robust cross-sample comparison, label-free quantification (LFQ) intensities from the global proteomic analysis were processed. The intensities were log_2_-transformed and normalized across all samples using a quantile normalization method. For the Kbhb-modified peptide dataset, a dedicated normalization pipeline was implemented to assess changes in modification stoichiometry independently of variations in parent protein abundance. First, the relative quantification value for each modified peptide in each sample was calculated by dividing its intensity by the mean intensity of that peptide across all samples (center-scale transformation). Subsequently, to correct for the influence of protein expression changes, these peptide-centric relative values were divided by the relative quantification value of their corresponding parent protein obtained from the global proteome analysis of the same sample. This yielded a protein-abundance-normalized value representing the modification level.

Differential expression analysis for proteins and differential abundance analysis for Kbhb-modified sites between the fed and 72 h starved groups were performed using Student’s *t*-test. For each protein or modified site, the fold change (FC) was calculated as the ratio of the mean normalized abundance between the two groups. The corresponding *p*-value was derived from the *t*-test. Statistical significance was defined by a nominal *p*-value < 0.05, combined with an absolute log_2_ fold change (|log_2_FC|) threshold of >0.585 (corresponding to FC > 1.5). This threshold combination is standard in discovery-phase proteomic and PTM studies to identify alterations of substantial magnitude for downstream functional analysis and hypothesis generation.

### 2.8. Bioinformatic and Structural Analyses

Subcellular localization annotations were curated from UniProt and grouped into nucleus, cytoplasm, mitochondria, and other compartments. Sequence motifs flanking ±7 amino acids around Kbhb sites were analyzed using motif-x and TwoSampleLogo to identify enriched residues. Functional pathway enrichment analyses (KEGG, GO) were conducted with clusterProfiler (v3.22) in R (adjusted *p*-value < 0.05).

Protein structural models were derived from AlphaFold predictions and experimentally solved structures within the Protein Data Bank (PDB). Kbhb sites, catalytic residues, and known post-translational modification hotspots were mapped in PyMOL (v3.1). Minimal atomic distances between Kbhb-modified lysines and catalytic/cofactor-binding residues were calculated. Evolutionary conservation scores were computed via MAFFT (v7) multiple sequence alignments and Rate4Site (v.2.01).

### 2.9. Statistical Analysis

Each experimental condition included four biological replicates. Data reproducibility was validated through principal component analysis (PCA), relative standard deviation calculations, and Pearson correlation. All quantitative physiological data are expressed as mean ± standard deviation (SD). Differences between groups were evaluated by unpaired Student’s *t*-test, with significance threshold set at *p* < 0.05. Data visualization was performed using GraphPad Prism 8 software.

### 2.10. Immunoprecipitation and Western Blot Analysis

Gastrocnemius muscles from control and 72 h starved mice were harvested and immediately snap-frozen in liquid nitrogen. Tissues were homogenized in ice-cold IP lysis buffer (50 mM Tris-HCl pH 7.4, 150 mM NaCl, 1 mM EDTA, 1% NP-40, 0.5% sodium deoxycholate) supplemented with protease inhibitor cocktail (Roche), phosphatase inhibitor cocktail (Roche), 5 mM nicotinamide (NAM), and 5 mM sodium butyrate to preserve protein modifications. The homogenates were incubated on ice for 30 min with occasional vortexing, followed by centrifugation at 12,000× *g* for 15 min at 4 °C. The supernatants were collected, and protein concentrations were determined using the BCA Protein Assay Kit (Thermo Fisher Scientific, Waltham, MA, USA).

For immunoprecipitation, 1 mg of total protein lysate was pre-cleared with Protein A magnetic beads for 1 h at 4 °C with gentle rotation. Pre-cleared lysates were then incubated with anti-SIRT3 antibody (Cell Signaling Technology, #5490, 1:100 dilution, Danvers, MA, USA) overnight at 4 °C with gentle rotation. The following day, 30 μL of Protein A magnetic beads were added and incubated for an additional 2 h at 4 °C. The beads were then washed five times with ice-cold IP lysis buffer. Bound proteins were eluted by boiling in 2× SDS-PAGE loading buffer at 95 °C for 10 min.

The immunoprecipitated samples and input controls were separated by 12% SDS-PAGE and transferred onto PVDF membranes (Millipore, St. Louis, MO, USA). Membranes were blocked with 5% non-fat milk in TBST (TBS containing 0.1% Tween-20) for 1 h at room temperature. The membranes were then incubated with primary antibodies overnight at 4 °C. The following primary antibodies were used: anti-SIRT3 (Cell Signaling Technology, #5490, 1:1000), anti-pan-Kbhb (PTM BioLab, PTM-1201, 1:1000, Hangzhou, China), and anti-β-Actin (Sigma-Aldrich, A5441, 1:5000, St. Louis, MO, USA). After washing with TBST, membranes were incubated with HRP-conjugated secondary antibodies (1:5000) for 1 h at room temperature. Protein bands were visualized using enhanced chemiluminescence (ECL) reagents (Thermo Fisher Scientific) and detected by a chemiluminescence imaging system.

## 3. Results

### 3.1. Starvation Induces Significant Body Weight Loss, Muscle Atrophy and Systemic Ketogenesis

**Figure 1:**
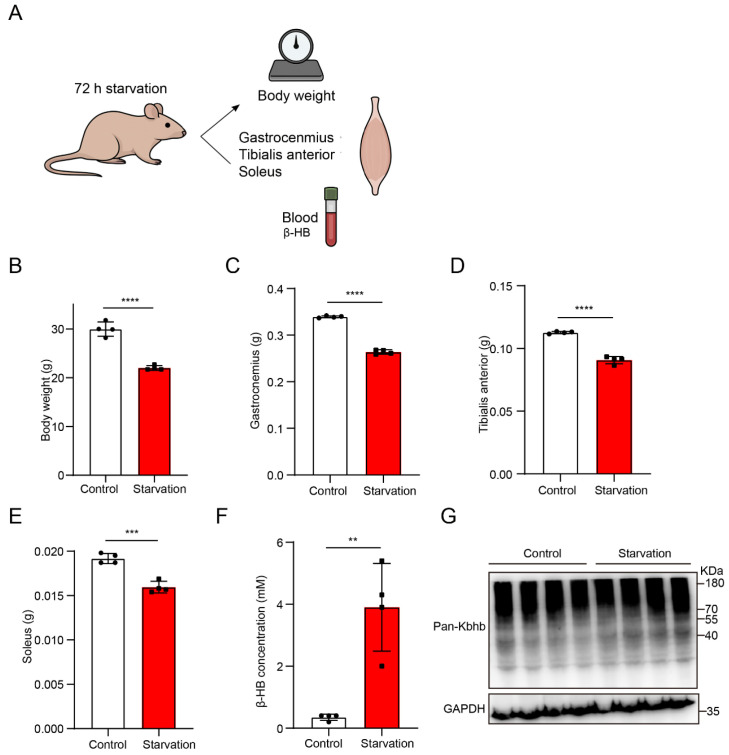
Starvation reduces body and muscle mass while increasing circulating β-hydroxybutyrate levels. (**A**) Schematic of the 72 h starvation protocol and sample collection, including body weight measurement, dissection of gastrocnemius, tibialis anterior, and soleus muscles, and collection of blood for β-HB analysis. (**B**–**E**) Body weight (**B**), gastrocnemius weight (**C**), tibialis anterior weight (**D**), and soleus weight (**E**) in control and starved mice. (**F**) Circulating β-HB concentrations showing a significant increase following starvation. (**G**) Representative immunoblot of pan-Kbhb in skeletal muscle from control and starved mice. Data are presented as mean ± SD. Statistical analyses were performed using unpaired two-tailed *t*-tests. ** *p* < 0.01; *** *p* < 0.001; **** *p* < 0.0001.

To establish a physiological starvation model and evaluate its impact on skeletal muscle homeostasis, mice were subjected to 72 h of food deprivation (Figure 1A). Starved mice exhibited a marked decrease in body weight (from 29.3 ± 0.7 g to 22.0 ± 0.5 g, representing a 24.9% loss) relative to ad libitum-fed controls (Figure 1B), confirming effective induction of systemic energy deprivation. Correspondingly, there was a significant reduction in the mass of key hindlimb muscles—including gastrocnemius (Figure 1C), tibialis anterior (Figure 1D), and soleus (Figure 1E)—reflective of acute muscle catabolic processes triggered by nutrient scarcity.

Metabolic adaptation to starvation was assessed by quantification of circulating β-hydroxybutyrate (β-HB), the predominant ketone body during prolonged fasting [25]. Starved mice showed a robust elevation in plasma β-HB levels (Figure 1F), indicative of enhanced hepatic ketogenesis and an increased reliance on ketone bodies as alternative energy substrates.

To validate the occurrence of lysine β-hydroxybutyrylation (Kbhb) in skeletal muscle, Western blotting was performed using a pan-Kbhb antibody. Multiple distinct Kbhb-immunoreactive bands were detected across the gastrocnemius proteome (Figure 1G), suggesting widespread β-hydroxybutyrylation in muscle proteins.

Collectively, these data demonstrate that 72 h starvation robustly induces systemic energy deficit, muscle atrophy, activation of ketogenesis, and pervasive Kbhb modification in skeletal muscle.

### 3.2. Global Proteomic Remodeling of Skeletal Muscle Triggered by Starvation

**Figure 2:**
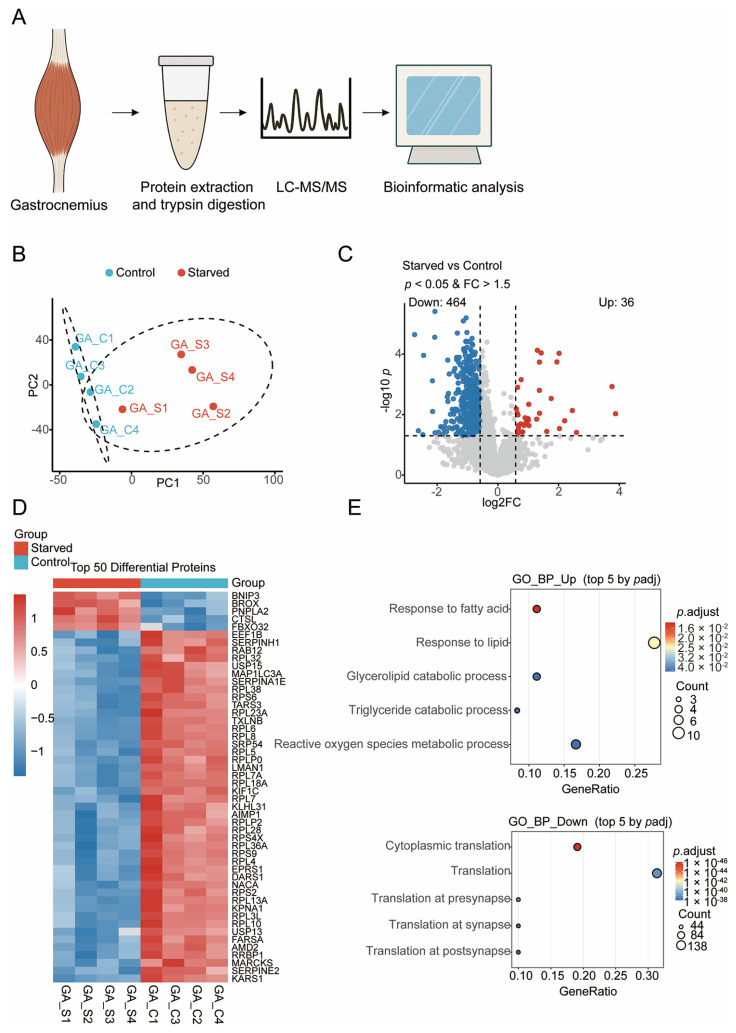
Global proteomic remodeling of skeletal muscle under starvation. (**A**) Schematic overview of the quantitative proteomic workflow. Gastrocnemius muscles from control and 72 h starved mice were collected for protein extraction, trypsin digestion, LC–MS/MS acquisition, and subsequent bioinformatic analyses. (**B**) PCA showing distinct clustering between control (blue) and starved (red) muscle samples. (**C**) Volcano plot of differentially expressed proteins, highlighting upregulated (red) and downregulated (blue) proteins. (**D**) Heatmap of the top 50 differential proteins. (**E**) GO enrichment analysis showing the top five biological processes for upregulated and downregulated proteins. Dot size represents the number of proteins, and color denotes adjusted *p*-value.

To delineate starvation-induced proteomic alterations, quantitative proteomic profiling was performed on gastrocnemius muscles from control and starved mice (Figure 2A). PCA revealed clear segregation between the two groups along the first principal component (Figure 2B), evidencing extensive proteome reprogramming in response to nutrient deprivation. The observed tight clustering within groups underscored dataset reproducibility and biological consistency.

Differential expression analysis identified 500 proteins exhibiting statistically significant changes, with 36 upregulated and 464 downregulated under starvation (Figure 2C). The volcano plot highlighted a pronounced downregulation trend, consistent with global suppression of anabolic pathways. Hierarchical clustering of the top 50 differentially expressed proteins distinctly segregated the two groups (Figure 2C).

Notably, starvation elevated protein abundance of BNIP3, CTSL, and PNPLA2, key regulators of autophagy, lysosomal proteolysis, and lipid mobilization, respectively, implicating enhanced organelle turnover and lipid catabolism [26]. Conversely, numerous ribosomal proteins (e.g., RPL3, RPL4, RPS6, RPL13A) and translation elongation factors (EEF1B, EPRS1) were markedly downregulated, reflecting coordinated inhibition of cytoplasmic translation and ribosome biogenesis [27]. This reciprocal expression signature is consistent with a shift from energy-intensive protein synthesis to catabolic processes supporting energy release and cellular maintenance.

Functional enrichment analyses corroborated these findings. Upregulated proteins were enriched in fatty acid catabolic processes, glycerolipid degradation, and oxidative stress responses (Figure 2E), corroborating a metabolic transition toward lipid utilization and redox homeostasis. Downregulated proteins were predominantly associated with translation, ribosomal assembly, and synaptic protein synthesis, supporting a broad suppression of protein biosynthesis machinery during starvation.

Thus, starvation orchestrates a proteomic signature characterized by activation of autophagy–lipid metabolic networks and repression of protein biosynthesis, constituting an adaptive strategy to conserve energy and maintain muscle homeostasis under nutrient restriction.

### 3.3. Comprehensive Atlas of Lysine β-Hydroxybutyrylation in Mouse Skeletal Muscle

**Figure 3:**
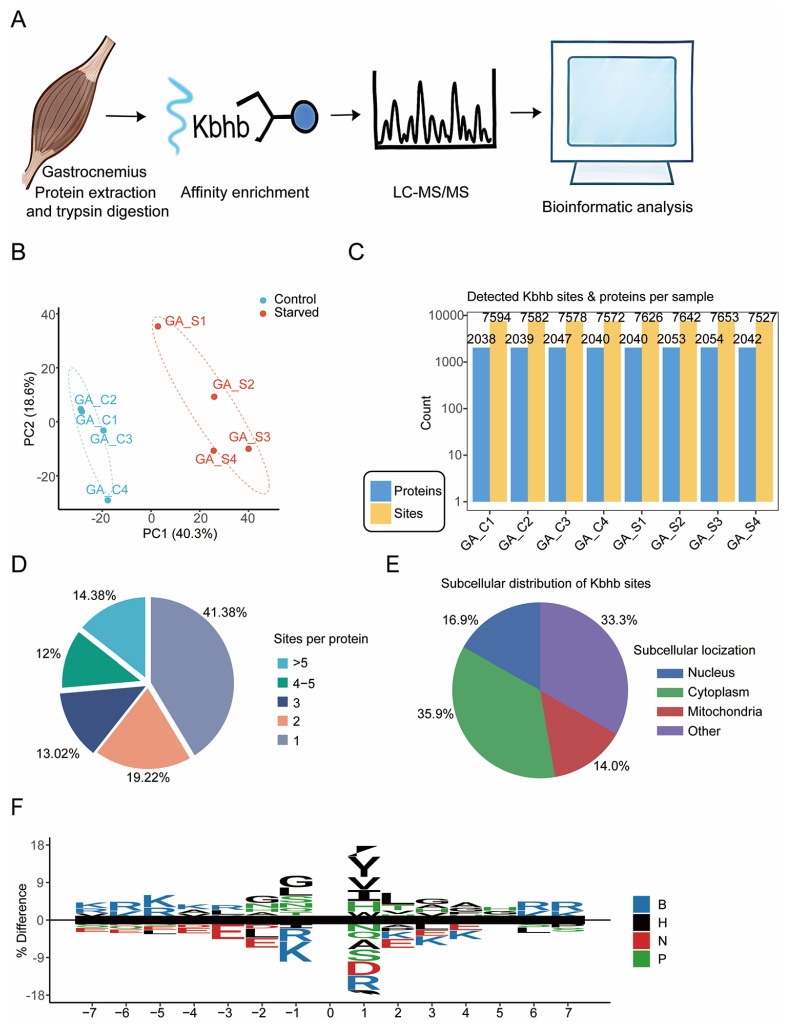
Global landscape of lysine β-hydroxybutyrylation (Kbhb) in mouse skeletal muscle. (**A**) Schematic workflow of the Kbhb proteomic analysis. Gastrocnemius muscles from control and 72 h starved mice were subjected to protein extraction and trypsin digestion, followed by Kbhb affinity enrichment, LC-MS/MS acquisition, and bioinformatic analyses. (**B**) PCA of all quantified Kbhb sites across biological replicates from control (GA_C) and starved (GA_S) gastrocnemius muscles. (**C**) Numbers of identified Kbhb sites and Kbhb-modified proteins across all samples. (**D**) Distribution of Kbhb site density per protein. (**E**) Subcellular localization of Kbhb-modified proteins. (**F**) Sequence motif of ±7 amino acids surrounding Kbhb-modified lysines. Amino acid categories are color-coded (B, basic; H, hydrophobic; N, negative; P, polar). Motif significance: *p* = 0.05.

To establish a comprehensive atlas of lysine β-hydroxybutyrylation (Kbhb) in skeletal muscle, we performed deep Kbhb proteomic profiling in gastrocnemius muscles from control and starved mice (Figure 3A). Across all samples, Kbhb peptides were quantified with high reproducibility, as demonstrated by PCA, which showed tight clustering within groups and clear separation between conditions (Figure 3B). These data confirm the robustness of our Kbhb enrichment and LC–MS workflow.

Deep proteomic coverage enabled the identification of more than 7500 Kbhb sites and approximately 2000 Kbhb-modified proteins in each sample (Figure 3C), which, to our knowledge, provides a broad and deep characterization of the skeletal muscle Kbhb landscape. Examination of site occupancy revealed that although a substantial proportion of proteins carried only a single Kbhb site (41.38%), the majority of modified proteins were multiply modified, with ~60% containing two or more Kbhb sites and ~14% carrying more than five sites (Figure 3D). This wide range of modification stoichiometry suggests heterogeneous regulatory potential across the Kbhb proteome.

Subcellular localization analysis showed that Kbhb-modified proteins are broadly distributed within skeletal muscle cells (Figure 3E). The largest fraction localized to the cytoplasm (35.9%), consistent with extensive modification of metabolic enzymes and cytoskeletal components. Additional modifications were found in the nucleus (16.9%) and mitochondria (14.0%), indicating potential roles in transcriptional regulation and mitochondrial metabolism. The remaining 33.3% mapped to other compartments, reflecting the widespread influence of Kbhb across cellular processes.

Motif analysis of amino acids flanking Kbhb sites revealed a distinct sequence preference (Figure 3F). Basic residues such as lysine and arginine were strongly enriched near the modified site, while hydrophobic residues (V, L, I) and acidic residues (D, E) appeared at defined positions. These patterns suggest that local electrostatic and structural features contribute to the selection or accessibility of Kbhb sites.

Collectively, these results provide a comprehensive landscape of Kbhb modification in skeletal muscle, revealing its extensive coverage, diverse subcellular distribution, and characteristic sequence features. This atlas establishes a foundation for understanding how Kbhb contributes to skeletal muscle physiology and metabolic adaptation.

### 3.4. Starvation Induces Widespread Remodeling of the Kbhb Proteome in Skeletal Muscle

**Figure 4:**
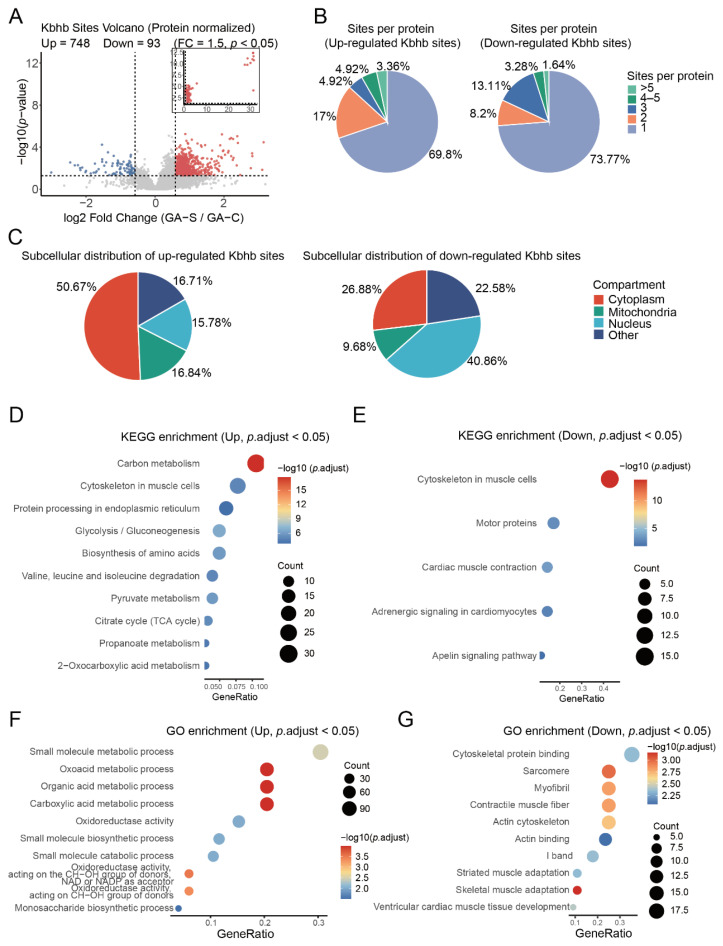
Starvation triggers global remodeling of lysine β-hydroxybutyrylation (Kbhb) in skeletal muscle. (**A**) Volcano plot showing differentially regulated Kbhb sites between starved (GA-S) and control (GA-C) muscles after intensity normalization, highlighting upregulated (red) and downregulated (blue) Kbhb sites. (**B**) Distribution of Kbhb site counts per protein for up-regulated (left) and down-regulated (right) sites. (**C**) Subcellular localization of up-regulated (left) and down-regulated (right) Kbhb sites. (**D**) KEGG pathway enrichment of up-regulated Kbhb sites (*p*.adjust < 0.05). (**E**) KEGG pathway enrichment of down-regulated Kbhb sites (*p*.adjust < 0.05). (**F**) GO enrichment of up-regulated Kbhb sites (*p*.adjust < 0.05). (**G**) GO enrichment of down-regulated Kbhb sites (*p*.adjust < 0.05).

Quantitative site-specific analysis revealed profound alterations in Kbhb levels during starvation, with 748 sites significantly upregulated and 93 downregulated (Figure 4A). The volcano plot demonstrates a global increase in Kbhb modifications under nutrient deprivation.

To further validate the upregulation of Kbhb identified by mass spectrometry, we performed immunoprecipitation followed by Western blot analysis using SIRT3 as a representative target. As shown in Supplementary Figure S1, we immunoprecipitated SIRT3 from gastrocnemius muscle lysates of control and 72 h starved mice, followed by Western blot analysis using pan-Kbhb antibody. The results demonstrated that the Kbhb signal associated with immunoprecipitated SIRT3 was markedly increased in starved mice compared to control mice. These results are consistent with our mass spectrometry findings and confirm that SIRT3 Kbhb modification is indeed upregulated upon starvation, supporting the reliability of our proteomic dataset.

Distribution analysis showed that upregulated Kbhb sites were mostly localized on proteins with a single modified site (69.8%), while multi-site modifications were less frequent (Figure 4B). Downregulated sites exhibited a comparable distribution pattern (Figure 4B), indicating a broad but targeted remodeling of Kbhb.

Subcellular localization analysis revealed functional preferences: upregulated Kbhb sites were enriched predominantly in cytoplasmic proteins (50.67%), followed by mitochondrial (16.84%) and nuclear (16.71%) proteins, whereas downregulated sites were distributed with less cytoplasmic predominance and relatively greater mitochondrial and nuclear representation (Figure 4C). This suggests starvation preferentially augments Kbhb on cytosolic metabolic enzymes while decreasing modifications on certain mitochondrial and nuclear proteins.

To characterize sequence features associated with starvation-responsive Kbhb sites, we performed separate motif analyses for upregulated and downregulated sites (Supplementary Figure S2). Upregulated Kbhb sites showed a preferential enrichment of acidic residues, particularly aspartic acid (D), at the +1 position relative to the modified lysine. In contrast, downregulated sites exhibited a distinct sequence pattern characterized by enrichment of arginine (R), with additional representation of glycine (G), at positions −5 to −3, as well as enrichment of tyrosine (Y) proximal to the modification site. These distinct sequence contexts suggest that the local amino acid environment is associated with differential Kbhb regulation during starvation, potentially reflecting differences in site accessibility or sequence-dependent regulation, although the underlying molecular mechanisms remain to be determined.

Pathway enrichment analysis of upregulated Kbhb proteins highlighted involvement in key metabolic processes, including carbon metabolism, muscle cytoskeleton organization, protein processing in the endoplasmic reticulum, glycolysis/gluconeogenesis, amino acid biosynthesis, pyruvate metabolism, and the tricarboxylic acid (TCA) cycle (Figure 4D). In contrast, downregulated Kbhb sites were enriched in pathways relating to muscle structural organization and contractile function, such as sarcomere organization, motor proteins, cardiac muscle contraction, and adrenergic signaling (Figure 4E). The selective decrease in Kbhb on contractile machinery likely reflects adaptive restructuring of muscle function during fasting.

Gene Ontology (GO) enrichment further substantiated these findings: upregulated Kbhb sites were enriched in small molecule metabolic processes, oxidoreductase activity, carboxylic acid metabolism, and monosaccharide biosynthesis (Figure 4F), while downregulated sites were linked to cytoskeletal protein binding, sarcomere structure, myofibril organization, and muscle adaptation mechanisms (Figure 4G).

Altogether, starvation precipitates a dual-layer Kbhb remodeling in skeletal muscle, characterized by enhanced modification of metabolic enzymes to support energy homeostasis and concurrent reduction in modifications on structural proteins to modulate contractile functions.

### 3.5. Starvation-Enhanced Kbhb Targets Functionally Critical Lysine Residues on Key Metabolic Enzymes

**Figure 5:**
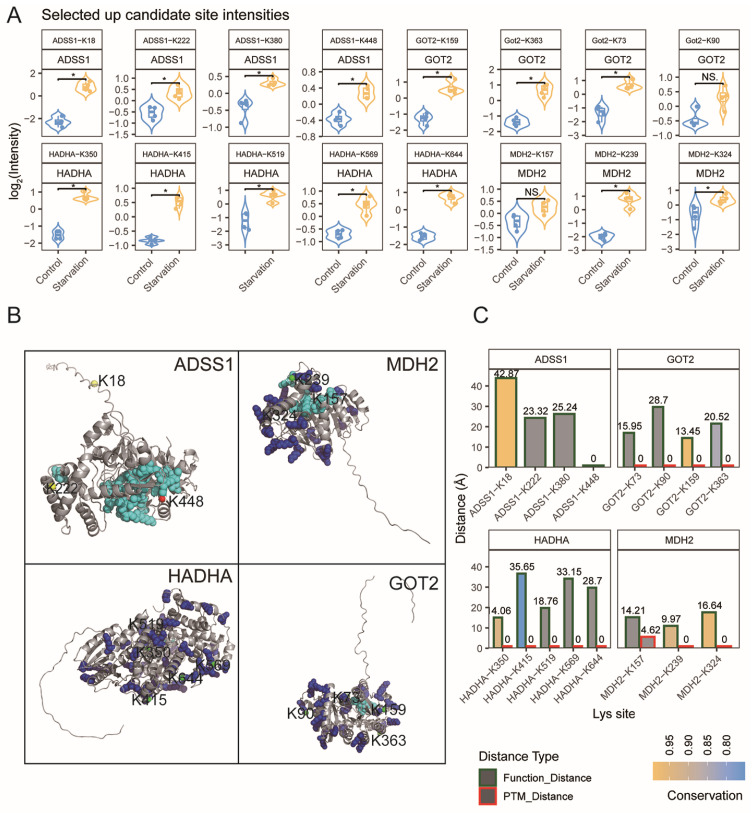
Structural and functional characterization of starvation-responsive Kbhb sites on key metabolic enzymes. (**A**) Quantitative intensities of representative up-regulated Kbhb sites identified from ADSS1, HADHA, GOT2, and MDH2. Violin plots show log_2_-normalized intensities in control and starved gastrocnemius muscle. NS.: not significant; * *p* < 0.05. (**B**) Structural mapping of representative key proteins highlighting the spatial distribution of Kbhb-modified lysines relative to functional and post-translational modification (PTM) hotspots. Protein structures are shown in grey, with functional catalytic or cofactor-binding residues and known PTM sites highlighted in distinct colors. Each lysine residue is rendered as a sphere with color coding based on its spatial relationship to functional/PTM sites: Orange: overlaps with a known PTM site and is within 5 Å of a functional site; Green: overlaps with a PTM site but not near functional sites; Red: does not overlap with PTM sites but lies within 5 Å of a functional site; Yellow: neither overlaps with PTM sites nor lies near functional sites. Lysine labels indicate residue numbers, illustrating their three-dimensional positions and potential regulatory relevance. (**C**) Spatial proximity and evolutionary conservation of key lysine residues. Bar plots show the minimum atomic distances from each lysine to the nearest functional residue and the nearest PTM site, together with conservation scores derived from multi-species sequence alignment.

To elucidate how starvation-induced Kbhb modulates metabolism, structural and functional analyses were conducted on representative Kbhb sites elevated upon starvation on enzymes pivotal to central metabolism. Quantitative proteomics confirmed significant increases in Kbhb at lysine residues on ADSS1, MDH2, HADHA, and GOT2—key enzymes involved in purine biosynthesis, the TCA cycle, fatty acid β-oxidation, and amino acid metabolism, respectively (Figure 5A).

Mapping of these sites onto known or predicted 3D protein structures demonstrated that starvation-responsive Kbhb sites occupy strategic functional domains. For example, structural mapping showed that ADSS1-K448 is situated approximately 14 Å from the GTP-binding pocket. Multiple Kbhb sites on MDH2 (K239, K324) and HADHA (K415, K569, K644) were found to spatially overlap with known lysine residues that are modified by other post-translational modifications (Figure 5B).

Integrative analysis combining evolutionary conservation and spatial proximity to catalytic or cofactor-binding residues prioritized ADSS1-K448, GOT2-K159, and MDH2-K239 as high-confidence allosteric or active site modifications, with conservation scores exceeding 0.85 (Figure 5C).

These insights reveal that starvation induces selective Kbhb enhancement at conserved, functionally critical lysines, suggesting β-hydroxybutyrylation functions as a dynamic metabolic regulator. Collectively, these structural features position these starvation-responsive Kbhb sites as potential candidates for the direct regulation of enzyme function, thereby augmenting ATP production and metabolic flexibility without necessitating genomic or translational changes.

## 4. Discussion

In this study, we systematically characterized proteomic and lysine β-hydroxybutyrylation (Kbhb) dynamics in skeletal muscle subjected to 72 h of starvation. Our data suggest that this multifaceted remodeling underpins metabolic adaptation and muscle homeostasis under acute nutrient deprivation. Our integrative analysis establishes starvation not only as a potent inducer of muscle proteome reprogramming but also as a trigger for widespread, site-specific Kbhb modification, highlighting β-hydroxybutyrylation as a potential regulatory mechanism coordinating metabolic flux and muscle structure–function balance.

The significant loss in body weight and skeletal muscle mass observed aligns with well-documented muscle atrophy induced by acute nutrient restriction [4,5]. Consistent with this phenotype, our quantitative proteomic analysis implicated extensive downregulation of ribosomal proteins and translation elongation factors, indicating a coordinated shutdown of protein biosynthesis machinery. This global translational repression is a classical hallmark of energy conservation during starvation [28,29]. Meanwhile, the upregulation of autophagy markers such as BNIP3 and lysosomal proteases like CTSL corroborates enhanced protein turnover and organelle recycling, aligned with increased catabolic flux essential for maintaining cellular homeostasis during metabolic stress [30,31].

The observed elevation in proteins involved in lipid catabolism, notably PNPLA2, reflects a metabolic switch typical of fasting, wherein skeletal muscle increases fatty acid mobilization and oxidation to compensate for glucose scarcity [7,32]. Enrichment of pathways related to fatty acid degradation and oxidative stress responses further confirms this adaptive metabolic reprogramming toward enhanced reliance on lipids and ketone bodies as primary fuel sources [8,33]. Together, these proteomic shifts depict skeletal muscle as a metabolically flexible tissue that suppresses energetically costly anabolic programs while activating catabolic and oxidative metabolic routes during starvation.

Our deep proteomic profiling implicates that Kbhb is a pervasive PTM in skeletal muscle, with over 7500 sites on ~2000 proteins, including metabolic enzymes, structural proteins, and transcription-related factors. This comprehensive atlas significantly extends previous catalogs of histone and non-histone Kbhb sites [19,34,35], suggesting extensive modification diversity in a physiologically relevant tissue under fasting stress.

Strikingly, starvation induced a global increase in Kbhb, predominantly on proteins localized in the cytoplasm and mitochondria. This underscores the potential impact of this PTM on metabolic enzyme regulation [19,36]. We observed a preferential enrichment of Kbhb on enzymes involved in glycolysis, the TCA cycle, fatty acid oxidation, and amino acid metabolism. This pattern underscores β-hydroxybutyrylation as a putative metabolic switch that is responsive to elevated circulating β-hydroxybutyrate [8,37,38]. Meanwhile, decreased Kbhb on sarcomeric and cytoskeletal proteins suggests a functional reprioritization away from contraction toward metabolic adaptation—a notion supported by previous observations of muscle structural remodeling during starvation [39,40].

The motif enrichment around Kbhb sites, particularly the prevalence of basic residues adjacent to modified lysines, suggests recognition by specific modifying enzymes or readers may be mediated by local electrostatic interactions, reminiscent of other lysine acylations such as acetylation and succinylation [11,41,42]. These sequence features may underlie substrate specificity and functional targeting within the muscle proteome.

Functional and structural analyses indicate that increased Kbhb under starvation preferentially targets conserved lysines proximal to catalytically important sites within key metabolic enzymes such as ADSS1, MDH2, HADHA, and GOT2 [43,44]. The spatial mapping of these sites provides clues to their potential functional impact. For instance, ADSS1-K448 localizes in proximity to the GTP-binding pocket, where modification could sterically or electrostatically influence substrate docking. Similarly, residues like MDH2-K239 and HADHA-K415 lie at known regulatory hotspots for other acylations, suggesting Kbhb may participate in a combinatorial code to fine-tune activity. These spatial patterns support the hypothesis that starvation-induced Kbhb acts as a direct modulator of catalytic cores or allosteric networks. This pattern mirrors regulatory post-translational modifications known to modulate enzymatic function directly, including acetylation and succinylation [16,17,45]. For example, lysine modifications near the nucleotide-binding or catalytic domains may influence enzyme activity by sterically hindering substrate access or altering protein conformation [46,47]. Given the reversible nature of lysine acylations, Kbhb likely functions as a reversible mechanism for fine-tuning enabling rapid fine-tuning of metabolic flux independent of transcriptional changes, which is advantageous under fluctuating nutrient states [11,48].

These observations align with emerging evidence that β-hydroxybutyrate serves not only as a metabolic substrate but also as a signaling metabolite that can induce epigenetic and non-epigenetic protein modifications, thereby modulating cellular function and adaptation [8,49,50]. Our data thus contribute to this paradigm to skeletal muscle metabolic enzymes, positioning Kbhb as a critical player in starvation-induced metabolic plasticity.

Furthermore, the principles of metabolite-driven PTM regulation uncovered here—wherein a systemic metabolic shift (ketosis) instigates widespread protein modifications (Kbhb) to reconfigure enzymatic networks and tissue physiology—may extend beyond the context of nutrient stress. Similar molecular logic could underpin the optimization of complex traits in agricultural systems. For instance, in farm animals, heterosis (hybrid vigor) arising from crossbreeding (hybridization) often manifests as superior growth efficiency, feed utilization, and metabolic robustness—traits governed by intricate gene-regulatory and protein-interaction networks. Given that Kbhb dynamically remodels the activity landscape of core metabolic enzymes, we speculate that analogous PTM-mediated network tuning—a mechanism yet unexplored in this context—could contribute to the enhanced phenotypic performance in hybrids. Investigating whether hybridization in species like pigs, chickens, or cattle induces distinct, beneficial patterns of metabolic protein modifications (including but not limited to Kbhb) represents a compelling frontier, bridging fundamental metabolic regulation with agricultural genetics.

## 5. Limitations and Future Directions

While our study provides a comprehensive snapshot of proteome and Kbhb remodeling in response to acute starvation, several limitations remain. First, the functional consequences of individual Kbhb events on enzymatic activity and muscle physiology were not directly assessed. Furthermore, our mechanistic inferences based on structural modeling rely partly on predicted protein structures, and the potential for antibody cross-reactivity, as noted for some pan-Kbhb reagents, necessitates caution in interpreting enrichment data. Future studies employing site-directed mutagenesis, enzymatic assays, and in vivo functional perturbations are essential to validate mechanistic links between specific Kbhb modifications and metabolic outcomes. Second, the identification of enzymes responsible for Kbhb addition (“writers”) and removal (“erasers”) in skeletal muscle remains an open challenge. While histone lysine β-hydroxybutyrylation has been linked to the acetyltransferase activity of p300, while sirtuins may potentially serve as deacylases [44,51,52], the full repertoire and substrate specificity of these enzymes in muscle tissue warrant further exploration. Additionally, the dynamic interplay between Kbhb and other acyl modifications—such as acetylation, succinylation, and malonylation—remains to be elucidated in the context of nutrient stress. Crosstalk among these PTMs may orchestrate complex regulatory networks adapting metabolism and proteostasis. Moreover, future studies should include both sexes to elucidate any potential sex-dependent differences in proteomic and Kbhb responses to nutrient stress. As an exploratory, hypothesis-generating study, our primary aim was to provide a comprehensive landscape of the Kbhb proteome. The sample size (n = 4 per group), while sufficient to reveal large-scale changes, may limit the power to detect more subtle alterations.

The differential analysis of the proteomic and Kbhb-modificomic data, involving thousands of features, was conducted using a nominal *p*-value threshold (*p* < 0.05) combined with a stringent fold-change filter (|log_2_FC| > 0.585). We acknowledge that applying this approach without false discovery rate (FDR) correction in a high-dimensional dataset increases the risk of false positives. This choice was made considering the exploratory nature of this study and its limited sample size (n = 4 per group). In such a context, stringent multiple-testing correction (e.g., FDR < 0.05) can be overly conservative, potentially obscuring biologically relevant but moderately significant changes that are central to generating novel hypotheses. Our strategy of prioritizing large-magnitude changes (high fold-change) aimed to balance sensitivity with specificity. Nonetheless, we recognize this as a methodological limitation. Future studies with larger cohorts will be essential to apply more stringent correction methods and validate the identified targets with greater statistical confidence.

Expanding the analysis to chronic starvation, exercise, or disease models like cachexia and diabetes could illuminate the broader physiological and pathological roles of Kbhb. Furthermore, investigating whether modulation of Kbhb levels influences muscle atrophy or metabolic health could reveal novel therapeutic avenues.

As a speculative extension inspired by our findings, a promising translational extension of this work lies in agricultural science. The integrated proteomic-PTM profiling framework established here could be applied to investigate the molecular basis of heterosis in key farm animals (e.g., swine, poultry, cattle). Future studies could analyze skeletal muscle and other metabolically active tissues from purebred parental lines and their F1 hybrids to determine if superior hybrid performance in traits like growth rate, feed efficiency, and meat quality correlates with distinct, advantageous configurations of the protein modification landscape, including acylations like Kbhb. Such research would not only uncover novel mechanisms of hybrid vigor but also identify potential biomarkers or regulatory nodes for precision breeding strategies.

## 6. Conclusions

In summary, this study elucidates the extensive remodeling of the skeletal muscle proteome and lysine β-hydroxybutyrylation landscape during acute starvation, revealing coordinated suppression of anabolic processes and activation of catabolic and oxidative pathways. Our findings position Kbhb as a dynamic, metabolically sensitive PTM targeting conserved lysines on key metabolic enzymes, likely fine-tuning enzymatic activities to optimize energy production and substrate flexibility under nutrient scarcity. This work advances the understanding of metabolite-driven PTM mechanisms in muscle physiology and opens new directions for research into nutritional regulation and potential metabolic interventions.

## Data Availability

The mass spectrometry proteomics data, including all raw files and processed search results for both the global proteome and the Kbhb-enriched datasets, have been deposited to the ProteomeXchange Consortium (https://proteomecentral.proteomexchange.org) via the iProX partner repository with the dataset identifier IPX0014483000 (URL: https://www.iprox.cn//page/project.html?id=IPX0014483000, accessed on 21 January 2026).

## Supplementary Materials

The following supporting information can be downloaded at: https://www.mdpi.com/article/10.3390/biology15030289/s1, Figure S1. Validation of SIRT3 β-hydroxybutyrylation upregulation in starved mouse skeletal muscle. Representative Western blot images of SIRT3 immunoprecipitation from gastrocnemius muscles of control and 72-h starved mice. Input samples were probed with anti-SIRT3 and anti-Actin antibodies. Immunoprecipitated SIRT3 was probed with anti-pan-Kbhb antibody to detect β- hydroxybutyrylation levels. Figure S2. Motif analysis of differentially regulated Kbhb sites in response to starvation. Amino acid categories are color-coded (B, basic; H, hydrophobic; N, negative; P, polar). Motif sig-nificance: *p* = 0.05.
