# Supplementary material for: Comprehensive Proteomics and β-Hydroxybutyrylation Profiling in Starvation-Induced Gastrocnemius Muscle Remodeling
Source: Biology (Basel). 2026 Feb 6;15(3):289. doi: 10.3390/biology15030289 (PMC12897031; doi:10.3390/biology15030289)
Supplement: Supplementary file 1 [file biology-15-00289-s001.zip › biology-4053993-supplementary materials.pdf]

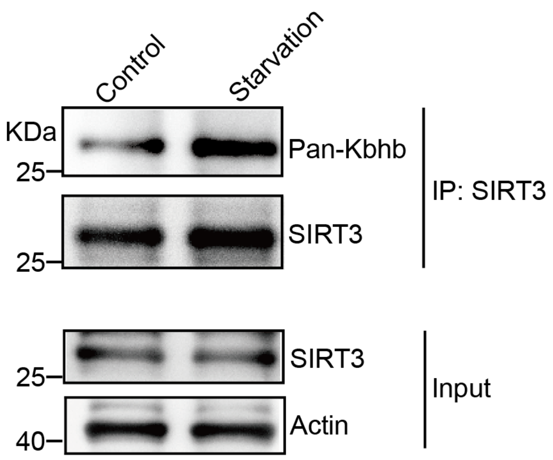

Supplementary figure 1. Validation of SIRT3 β-hydroxybutyrylation upregulation in starved mouse skeletal muscle. Representative Western blot images of SIRT3 immunoprecipitation from gastrocnemius muscles of control and 72-hour starved mice. Input samples were probed with anti-SIRT3 and anti-Actin antibodies. Immunoprecipitated SIRT3 was probed with anti-pan-Kbhb antibody to detect β-hydroxybutyrylation levels.

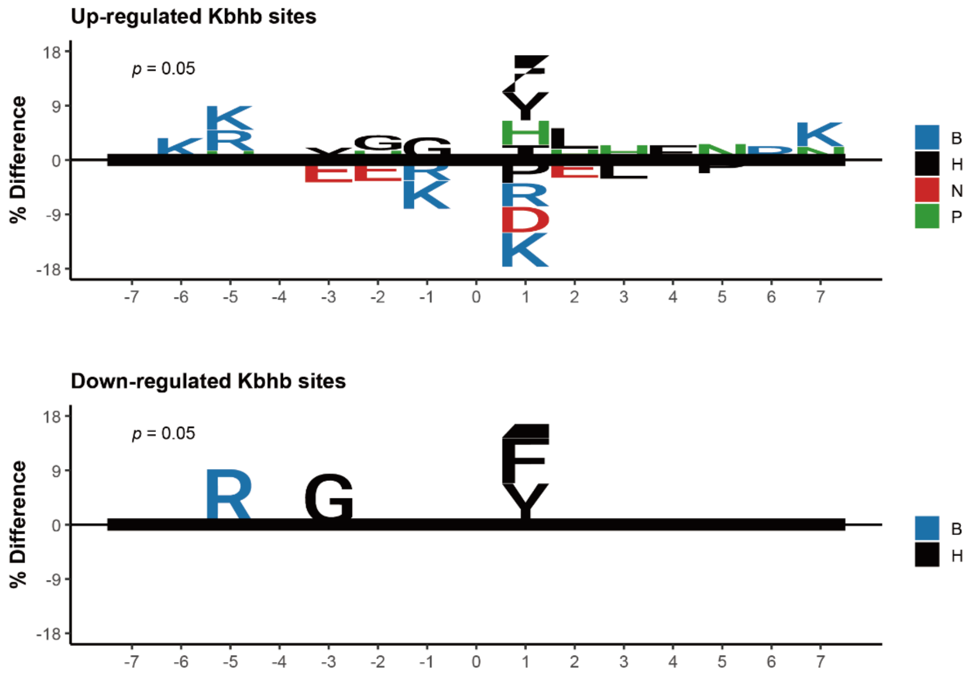

Supplementary Figure 2. Motif analysis of differentially regulated Kbhb sites in

response to starvation. Amino acid categories are color-coded (B, basic; H, hydrophobic; N, negative; P, polar). Motif sig-nificance: $p = 0.05$.
