# Supplementary figures and images for: Comprehensive Proteomics and β-Hydroxybutyrylation Profiling in Starvation-Induced Gastrocnemius Muscle Remodeling
Source: Biology (Basel). 2026 Feb 6;15(3):289. doi: 10.3390/biology15030289 (PMC12897031; doi:10.3390/biology15030289)

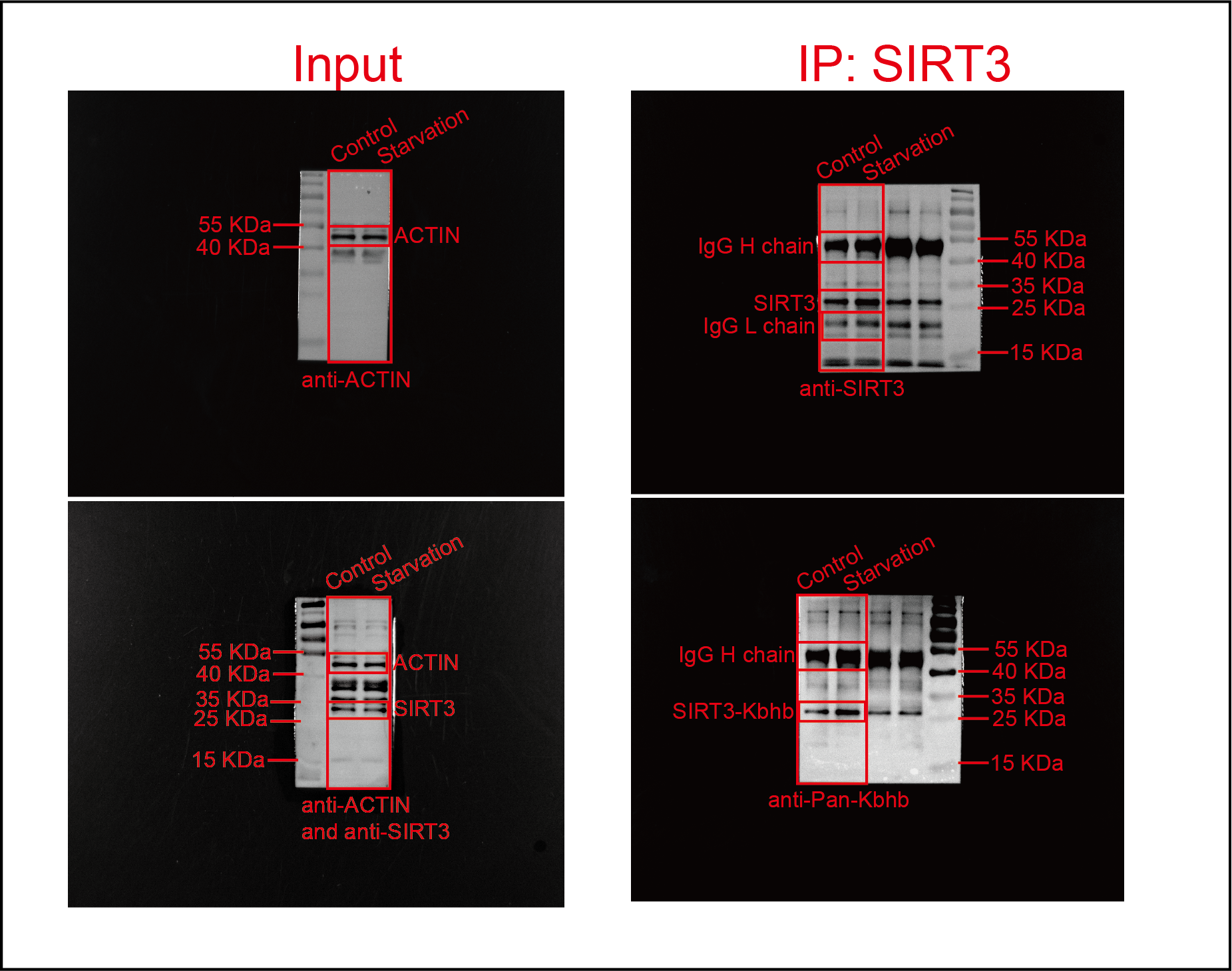

Supplement: Supplementary file 1 [file biology-15-00289-s001.zip › Western Blot Images of Figure 1.tif]
